# Attention-Deficit/Hyperactivity Disorder Symptoms Are Common and Associated with Worse Glycemic Control in Adults with Type 1 Diabetes

**DOI:** 10.3390/jcm14103606

**Published:** 2025-05-21

**Authors:** Yanli Zhang-James, Dan Draytsel, Ben Carguello, Stephen V. Faraone, Ruth S. Weinstock

**Affiliations:** 1 Department of Psychiatry and Behavioral Sciences, Norton College of Medicine, SUNY Upstate Medical University, 750 East Adams Street, Syracuse, NY 13210, USA; 2 Norton College of Medicine, SUNY Upstate Medical University, 750 East Adams Street, Syracuse, NY 13210, USA; draytsed@upstate.edu (D.D.);; 3 Department of Neuroscience and Physiology, Norton College of Medicine, SUNY Upstate Medical University, 750 East Adams Street, Syracuse, NY 13210, USA; 4 Department of Medicine, Norton College of Medicine, SUNY Upstate Medical University, 750 East Adams Street, Syracuse, NY 13210, USA; weinstor@upstate.edu

**Keywords:** Adult ADHD Self-Report Scale (ASRS), attention-deficit/hyperactivity disorder, cardiometabolic disease, type 1 diabetes mellitus

## Abstract

**Objective:** to assess the association between attention-deficit/hyperactivity disorder (ADHD), type 1 diabetes mellitus (T1D), and cardiovascular comorbidities in adults. **Methods:** The Adult Self-Report Scale V1.1 (ASRS) for ADHD symptoms was electronically sent to 2069 adults with T1D. Cardiometabolic conditions, laboratory measurements, and PHQ-2/PHQ-9 depression scores were obtained from the electronic medical record. **Results:** In total, 292 (14.1%) individuals responded and 279 consented to medical records extraction. The average age was 47.4 years (SD: ±18.9), 64.2% were women, 95.7% were non-Hispanic white, and the mean HbA1c level was 7.7% (±1.5%). Of 273 completing ASRS, 87 med ADHD criteria (ASRS positive, 31.9%), and 42 (15.4%) had an ADHD diagnosis or medication. Women had higher scores than men. ADHD symptoms decreased with age, but remained significantly higher than the general population levels. HbA1c levels were positively associated with the ASRS scores (Spearman’s r = 0.28, *p* < 0.0001). ASRS positive individuals had worse glycemic control (HbA1c ≥ 8.0%, adjusted OR 2.3, 95%CI: 1.3–4.1, *p* < 0.0001) and higher PHQ-9 scores (10 ± 7.3 vs. 6.1 ± 6, χ^2^_(1)_ = 9.2, *p* = 0.002) than the ASRS negative group. No associations were found between ASRS scores and cardiometabolic diseases, or other laboratory or clinical measurements. **Conclusions:** Many adults with T1D exhibit undiagnosed ADHD symptoms, which correlate with poorer glycemic control and depression. Further research with larger samples is needed to investigate ADHD prevalence and impacts in this group.

## 1. Introduction

Attention-deficit/hyperactivity disorder (ADHD) affects 5% of the population worldwide [1]. According to the International Diabetes Federation [2], 8.75 million people live with type I diabetes (T1D) worldwide in 2022. Both T1D and ADHD frequently have an onset in childhood and adolescence, but persist into adulthood. T1D is among the most frequently reported comorbid metabolic disorders in children with ADHD [3]. A systematic review and meta-analysis reported a 37% increase in the prevalence of T1D in children with ADHD compared to those without ADHD [4]. Large-scale population-based studies found that children born to mothers with T1D had increased risk of developing ADHD [5,6]. The polygenetic risk score for ADHD was found to be significantly associated with type 2 diabetes and other cardiometabolic conditions [7]. Glycemic dysregulation in T1D has been shown to impair executive functioning [8], and may contribute to long-term structural and functional alterations in the developing brain (Mauras, 2021) [9].

Conversely, the presence of ADHD symptoms can interfere with adherence to complex T1D treatment plans, which include monitoring glucose levels, administering multiple daily insulin injections or using an insulin pump, meal planning, and regularly engaging in physical activity, predisposing those with ADHD to worse outcomes, potentially including an increased risk of diabetes-associated cardiovascular comorbidities [10]. A few studies have reported higher levels of HbA1c, and more acute complications, such as diabetic ketoacidosis (DKA), severe hypoglycemia, and emergency room admissions, in those with a dual diagnosis of ADHD and T1D compared to those without ADHD [11,12,13]. One study reported that treating and managing ADHD symptoms with pharmacological therapy in people with T1D helped to improve glucose control and short-term complications [13]. Most of the literature in this field, however, focused on children, adolescents, or young adults [14,15,16], with relatively few studies examining adults [10,17], despite the fact that 82% of people living with T1D are ≥20 years of age [18]. ADHD symptoms also often persist into adulthood [19], and may go unrecognized in adults with T1D; thus, it is important to assess and understand the impact of ADHD symptoms in this population.

The goal of our current study was to assess ADHD symptoms among our adult patients with T1D, and examine if their ADHD symptom severity or dysfunction in the subcategories of executive dysfunction and emotional dysregulation were associated with cardiovascular comorbidities, elevated HgbA1c levels (worse glycemic control), elevated LDL-cholesterol and triglycerides (risk factors for cardiovascular disease), elevated alanine transaminase (ALT, a liver function test associated with fatty liver), or reduced kidney function (low eGFR, estimated glomerular filtration rate).

## 2. Methods

### 2.1. Sample

We identified individuals with a diagnosis of T1D (ICD-10-CM codes E10.XX), aged 18 years or older, who were receiving diabetes care at the Joslin Diabetes Center at Upstate Medical University in Syracuse, NY, from 1 January 2020 to 31 May 2022 from the electronic medical record (EHR; Epic Systems Corporation, Verona, WI, USA). They were contacted via email to complete surveys in REDCap (Vanderbilt University, Nashville, TN, USA). Consent for completion of the surveys and for additional data (described below) extracted from the Epic electronic medical record were obtained electronically. All data were deidentified for analysis. This work was approved by the Institutional Review Board for the Protection of Human Subjects of SUNY Upstate Medical University.

### 2.2. Assessments

As described in our previous study of adults with type 2 diabetes (T2D) [20], we used the Adult Self-Report Scale, V1.1 (ASRS) expanded version, to assess the DSM-5 symptoms of ADHD (nine symptoms for inattention and nine for hyperactivity/impulsivity), nine additional symptoms of executive dysfunction, and four symptoms of emotional control. Symptoms were scored following Adler et al.’s [21] guidelines, and we defined ASRS as Positive if there were five or more inattention symptoms, or five or more hyperactivity/impulsivity symptoms, according to the DSM-5 [22], and consistent with our previous study [20].

Data extracted from the Epic electronic medical record, including age (years), sex, race/ethnicity, insurance type (grouped by commercial/private, government-administered including Medicare and Medicaid, and managed care, as well as all others which include Tricare, Worker’s Comp, and unknown/uninsured), body mass index (BMI ≥ 30 kg/m^2^ defined obesity), smoking status, last recorded Patient Health Questionnaire PHQ-2 and PHQ-9 scores, and laboratory results for HbA1c, LDL-cholesterol, triglycerides, ALT, creatinine, and estimated glomerular filtration rate (eGFR), were analyzed in this study. PHQ9 was only administered when the PHQ2 score was ≥3.

Diagnostic history of ADHD (ICD-10-CM codes F90.X), obesity (E66), hypertension (I10–I15), cardiovascular diseases (peripheral artery/vascular diseases/arteriosclerosis, acute coronary syndrome, ischemic heart disease, deep vein thrombosis, pulmonary embolism, atrial fibrillation, supraventricular tachycardia, ventricular tachycardia, cardiac arrest, any arrhythmia as a broad term, any heart disease/heart failure, subarachnoid bleeding, hemorrhagic stroke, acute myocardial infarction, and ischemic stroke) were extracted from the problem list and medical history fields in Epic. Obesity was defined by either the presence of ICD-10-CM code E66, or ≥BMI 30 kg/m^2^. ADHD medications (including methylphenidate, amphetamine, guanfacine, clonidine, atomoxetine, and viloxazine) and insulin use were obtained from Epic. The presence of socioeconomic stress in the past 12 months was also recorded for each patient, including financial strain, and housing, transportation, and food insecurity (see the complete list of variables defined in the Abbreviations).

### 2.3. Statistical Analyses

We used the Pearson chi-square test for binary or categorical variables to evaluate associations (race, ethnicity, smoking history, presence of socioeconomic stress, and health insurance types) with the ASRS groups (ASRS positive vs. negative). Unpaired student *t*-tests were used to compare age and BMI between the ASRS groups. Negative binomial regression was used to model symptom scores such as ASRS, PHQ-2, and PHQ-9, adjusting for relevant demographic variables. This approach was appropriate due to the count-based nature and non-normal distribution of these symptom measures, which range from zero to their respective maximum scores. Logistic regression with the demographic covariates was used to assess the association of ASRS positive or negative status with cardiometabolic diagnoses.

A gamma generalized linear model (GLM) with demographic covariates was used to assess the relationship between ASRS grouping, scores, or ADHD diagnosis and medication use, with laboratory measurements treated as continuous outcomes. The gamma distribution with a log link was chosen due to the non-negative and positively skewed, continuous nature of many laboratory values (e.g., HbA1c). We also created clinically informed binary or categorical variables to indicate laboratory values that were outside of the normal range. HbA1c levels ≥8.0% were considered “High-HbA1c”, ALT values above 56 U/L were considered “high-ALT”, eGFR below 60 mL/min/1.73 m^2^ were considered “low-eGFR”, LDL cholesterol ≥130 mg/dL were “High-LDL”, and triglycerides ≥200 mg/dL were “High-TG”. Creatinine “High” was defined by values above the normal range of 0.74 to 1.35 mg/dL for men and 0.59 to 1.04 mg/dL for women. For these categories, we used a chi-square test to evaluate their associations with ASRS groups. Spearman correlation was used to assess the relationship between ASRS score and HbA1c levels.

An alpha level of 0.05 was used to determine statistical significance across all models, with adjustments for multiple comparisons made using the Bonferroni correction method.

## 3. Results

### 3.1. ASRS ADHD Symptom Scores

A total of 2069 individuals with T1D were contacted to complete the ASRS survey. Among them, 292 responded (14.1%). Among the responders, 279 (95.5%) consented for data extraction from the electronic medical record. Overall, mean age of the final cohort was 47.4 ± 18.9 years, 64.2% were women, and 95.7% were non-Hispanic white, with an average HbA1c level of 7.7 ± 1.5%. Among consented responders, six did not complete the ASRS survey and were excluded.

Among the 273 who completed the survey fully and had clinical data, 87 (31.9%) met ASRS symptom criteria for ADHD (“ASRS positive”), and 14 (5.1%) had a record of an ICD10 diagnosis of ADHD (10 ASRS positive and 4 ASRS negative respondents); a total of 42 respondents (15.8%) had either a diagnosis of ADHD or were taking ADHD medications (including 24 ASRS positive and 18 ASRS negative respondents). Significantly higher percentages of ASRS positive respondents had a diagnosis of ADHD, and/or taking ADHD medication. Table 1 list the summary of the ASRS scores, as well as participant characteristics, are shown in Table 1. Both ASRS positive and negative respondents were mostly non-Hispanic white. Although the racial, ethnic, and gender compositions, and insurance types, as well as the proportion of people who smoked, were not significantly different between the ASRS positive and negative groups, the symptom-positive group was overall 15.7 years younger than the negative group (mean ± standard deviation, SD: 36.8 ± 14.3 vs. 52.5 ± 18.3 years, z = 7.0, *p* < 0.001). The ASRS positive group had a significantly higher proportion (18.4%) of individuals who had at least one of the recorded socioeconomic stresses than the negative group (7.5%). The average BMI was similar between the groups.

**Figure 1:**
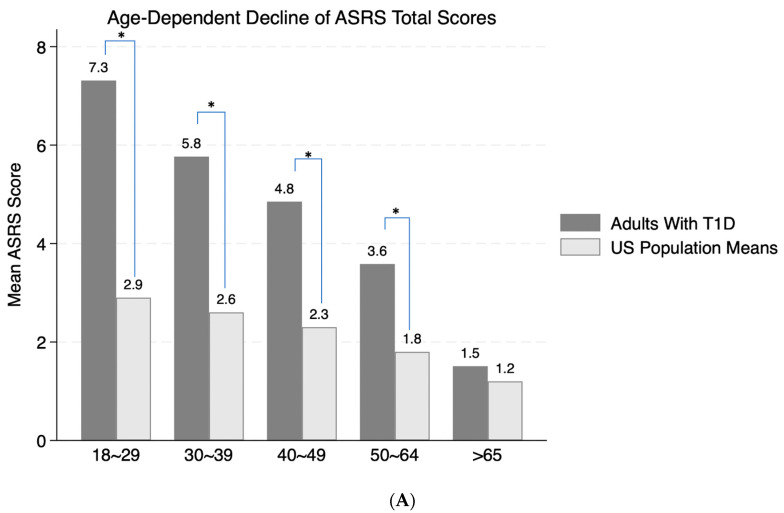

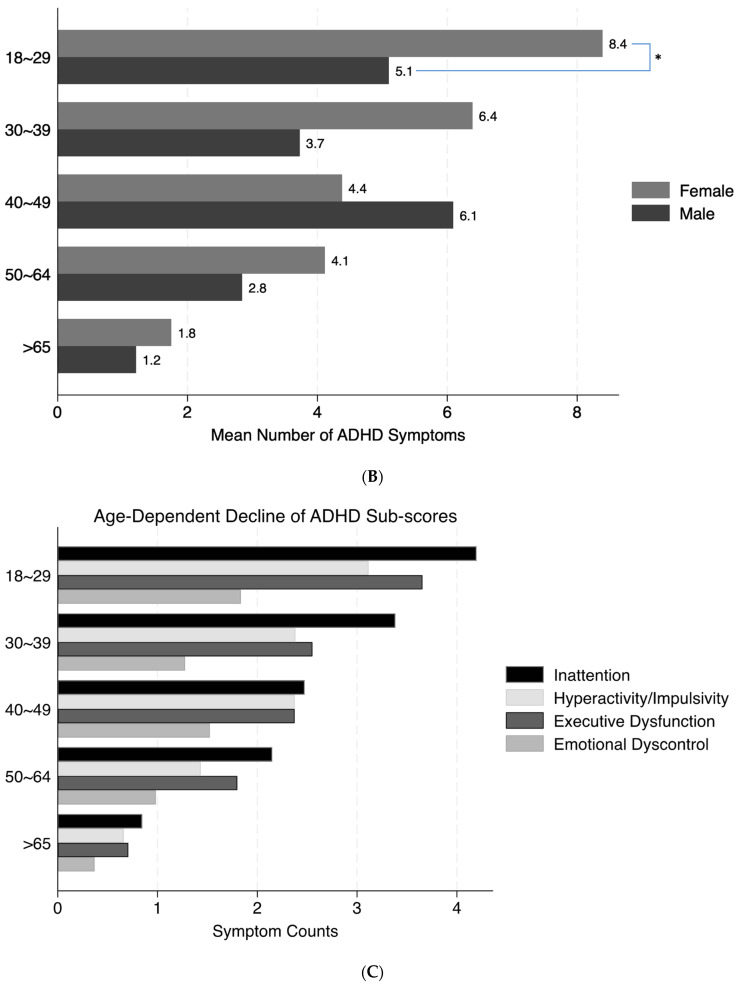

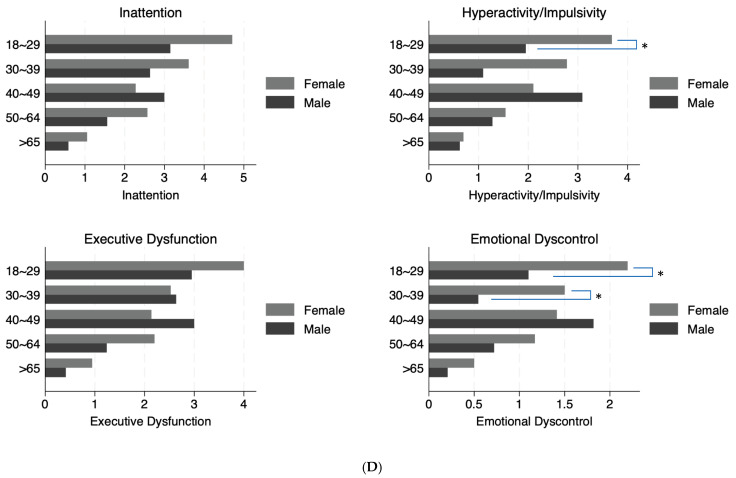
(**A**) Age-dependent decline of ASRS total scores, in contrast to the US population means. **Note:** Population means were based on a normative sample of 22,397 adults in the USA [23]. * indicates statistically significant difference within each age strata. (**B**) Gender difference of the age-dependent decline. * indicates statistically significant difference for the youngest age group (18–29 years old). (**C**) Age-dependent decline of ADHD sub-scores. (**D**) Gender difference of age-dependent decline of ADHD sub-scores. * indicates statistically significant difference for the youngest age group (18–29 years old).

There was a significant age-dependent decline of the ASRS-reported ADHD symptoms (χ^2^_(1)_ = 55.0, *p* < 0.0001; Figure 1A). Compared to normative data from 22,397 adults in the US population [23], as shown in Figure 1A, adults with T1D reported significantly higher symptom scores than those reported in the normative sample. For all age strata except those > 65 years, the normative population values were below the lower bound of the 95% confidence intervals of ADHD total symptom scores in our adults with T1D (indicated by * in Figure 1A). Overall, women reported significantly higher symptom counts than men (χ^2^_(1)_ = 4.4, *p* = 0.04), particularly in the younger groups (ages 18–29, highlighted by * in Figure 1B). The proportions of men and women across different age strata were similar (χ^2^_(4)_ = 7.7, *p* = 0.1), with more women than men across all age strata. There was no significant interaction between age and sex on the total symptom scores. All ADHD sub-scores also demonstrated significant age-dependent decline (Figure 1C), with consistently higher symptom counts for women than men overall. However, only women in the younger age group reported significantly higher symptom counts for hyperactivity/impulsivity and emotional dyscontrol (indicated by * in Figure 1D). Nevertheless, all subgroup comparisons were not significant after Bonferroni corrections.

Interestingly, there were no significant differences for all ASRS scores between those who had and those who never had a diagnosis of ADHD. There was some evidence suggesting that history of any ADHD medication prescription, however, was associated with higher ASRS total scores (marginal effect = 3.4, 95%CI: 0.4, 6.3; z = 2.2, *p* = 0.03), inattention (marginal effect = 2.0, 95%CI: 0.2, 3.7; z = 2.2, *p* = 0.03), hyperactivity/impulsivity (marginal effect = 1.4, 95%CI: 0.2, 2.6; z = 2.2, *p* = 0.03), and executive dysfunction (marginal effect = 1.6, 95%CI: 0.02, 3.3; z = 2.0, *p* = 0.047), but not emotional dyscontrol sub-scores. However, none of the above effects were significant after Bonferroni corrections.

### 3.2. Association of ADHD and Cardiometabolic Conditions

Table 2 lists the numbers and percentages of respondents with cardiometabolic diseases in ASRS positive vs. negative groups. Although the ASRS negative group had more individuals who have or had the diagnosis of most of the cardiometabolic conditions than the positive group, the statistical differences were not significant after adjusting for age, other demographic characteristics, socioeconomic stress, health insurance type, and smoking status, except for peripheral vascular diseases (PVD). Adjusted OR for PVD was 3.8 (95%CI: 1.2, 12.5; χ^2^_(1)_ = 5.2, *p* = 0.02) comparing the ASRS positive vs. negative groups. Similarly, we found some evidence of association between ASRS total scores and PVD (OR = 1.2, 95%CI: 1.0, 1.3; χ^2^_(1)_ = 6.8, *p* = 0.009; Supplementary Table S1). However, both *p*-values were not significant after the Bonferroni correction. There was no significant association between any other ASRS symptom scores and diagnosis of any cardiometabolic conditions (Supplementary Table S1).

Examining ADHD diagnostic status found evidence that people with diagnosed ADHD had higher risks of overall cardiovascular diseases (adjusted OR = 4.3, 95%CI: 1.3, 14.2; χ^2^_(1)_ = 5.5, *p* = 0.02) and PVD (OR = 7.4, 95%CI: 1.9, 28.5; χ^2^_(1)_ = 8.4, *p* = 0.004) compared with those never had a diagnosis, but both *p*-values were not significant after the Bonferroni corrections. There was no significant association between ADHD diagnosis and any other disorders. Whether individuals took ADHD medication or not was not associated with any cardiovascular diagnosis.

### 3.3. Laboratory and Mental Health Measures

Table 3 shows the mean and standard deviation (SD) of laboratory measurements and PHQ scores, as well as the numbers and proportions of abnormal laboratory values. We found that the mean HbA1c levels were similar between the ASRS positive and negative groups. However, a significantly higher proportion of individuals in the ASRS positive group had elevated A1c levels (HbA1c ≥ 8.0%) than those in the negative group (52.9% vs. 25.4%, *p* < 0.0001). ASRS positive individuals were more than twice as likely to have elevated HbA1c, indicative of poor glycemic control (HbA1c ≥ 8.0%, adjusted OR 2.3, 95% CI 1.3, 4.1, *p* < 0.0001), with *p*-values remaining significant after the corrections. Furthermore, we found that HbA1c levels were positively correlated with ASRS total scores (Spearman’s r = 0.28, *p* < 0.0001, Supplementary Figure S1). ADHD diagnosis and medication usage were not associated with HbA1c levels or high-A1c status.

We found significantly higher PHQ-9 scores (more depressive symptoms) in the ASRS positive group than those in the ASRS negative groups, with *p*-values remaining significant after the corrections (Table 3). Higher ASRS total score was also associated with a significant increase in PHQ-9 scores (margins = 0.7, 95%CI: 0.2, 1.2; χ^2^_(1)_ = 11.7, *p* = 0.001, significant after correction; Supplementary Table S2). Other sub-scores were not associated with PHQ-9 scores.

There was a small but significant difference in the creatinine levels between the ASRS groups. However, the means of both groups were within the clinically normal range. In addition, the proportions of individuals who had abnormally high levels of creatinine were not significantly different between the groups after the corrections (Table 3). ASRS total and sub-scores were not significantly associated with creatinine levels, eGFR, or other laboratory measurements. No difference was found for any of the measurements between those who had and those who never had a diagnosis of ADHD. History of ADHD medication was associated with a small, but statistically significant, increase in creatinine (margins = 0.24, 95%CI: 0.06, 0.4; χ^2^_(1)_ = 9.3, *p* = 0.002, significant after correction), but had no relationship with any other measurements.

## 4. Discussion

In this study, we found that a significant proportion of adults with T1D exhibited symptoms of ADHD, with 31.9% of respondents meeting diagnostic criteria based on the Adult Self-Report Scale (ASRS). Similarly to the general population, the number of reported ADHD symptoms decreased with age. However, adults with T1D continued to experience more ADHD symptoms across almost all age strata than the general population. We found that a significantly higher proportion of individuals in the ASRS positive group had elevated A1c levels (Hb A1c ≥ 8.0%) and PHQ-9 scores than those in the negative group. However, ADHD symptom scores and sub-scores, including executive dysfunction and emotional dyscontrol, were not associated with the diagnoses of any cardiometabolic diseases.

Compared to our previous study of adults with type 2 diabetes (T2D) [20], we had a slightly higher response rate in the current study of adults with T1D (14.1% vs. 10.5%), and a lower proportion of individuals who met the ASRS positive threshold (31.9% vs. 49.2%). These differences could be due to the demographic differences of the two patient cohorts. Adults with T1D in the current study were younger, and had more non-Hispanic and white females than the adults with T2D from our previous study [20]. Nevertheless, the elevated ASRS symptom scores compared to that of the general population, the age-dependent decrease of the symptom scores, and the lack of association with cardiometabolic diseases were similar for both studies of T1D and T2D, suggesting a common link between ADHD and diabetes, and highlighting the importance of ADHD screening and management in both types of diabetes.

Previous reports have shown that depression is more common in people with ADHD of all ages, including children [24], adolescents [25], college students [26], and adults [27,28,29]. Systematic reviews also suggested that comorbid diabetes and depression are common [30]. A meta-analysis estimated an almost four times increase in the prevalence of depression in adults with T1D compared with those without [31]. Studies have reported shared genetic factors underlying the comorbidities between ADHD and depression [32,33], as well as between ADHD and T1D [34,35]. All three disorders (T1D, major depressive disorder, and ADHD) co-aggregated within families, further suggesting shared genetics [36]. We have previously reported that depressive symptoms in T1D adults are associated with higher HbA1c levels and less glucose monitoring, suggesting that depression screening and treatment should be integrated into the management of adults with T1D [37,38]. Our current results suggest that this may be of particular importance in people with ADHD.

Poorly controlled T1D (HbA1c ≥ 8.0%) was associated with having more ADHD symptoms in our adult cohort. Other studies [12,39], including our recent meta-analysis [40], have also shown that comorbid ADHD was associated with higher blood glucose and HbA1c, and more diabetes-related complications in youth with T1D compared to those with only T1D. ADHD symptoms measured by Five-To-Fifteen or the Child Behavior Checklist (CBCL) were also reported to be positively associated with higher HbA1c levels in people with T1D [41,42]. Only limited studies have examined adults [17]. Our findings that the adults with positive ASRS scores were twice as likely to have poor glycemic control than those with negative scores emphasize that adults with T1D should also be targeted for ADHD symptom assessment and management. ADHD pharmacotherapies in youth with T1D and ADHD were reported to be associated with lower HbA1c and fewer hospitalizations than those with dual diagnoses but untreated [13]. Whether treatment of ADHD will lead to improved glycemic control in adults will require further investigation, given that only a small proportion of our cohort had ADHD medication and that we did not have more information on the duration or the course of their ADHD treatment.

Our findings have several important clinical implications. First, healthcare providers should consider assessing and addressing ADHD symptoms in adults with T1D, as a substantial proportion of individuals in this population may experience these symptoms. It is possible that adults with ADHD were more likely to have participated in our study. However, only 10 (11.5%) of the ASRS positive individuals had a diagnosis of ADHD, and 23 (26.4%) were prescribed ADHD medications. The overall rate of diagnosed ADHD was 5% in all of the responders, similar to the previously reported prevalence of ADHD in people with diabetes ranging from about 2% to 12% in different studies [11,12,39,43], but substantially lower than the rate that we observed based on ASRS scores. This suggests that the vast majority of individuals who met ADHD diagnostic criteria were unrecognized, undiagnosed, and untreated. This was similarly observed in our previous study of individuals with T2D [20].

Secondly, the age-related trends and gender differences in ADHD symptoms highlight the need for tailored approaches to assessing and treating ADHD symptoms. Notably, younger women with T1D were among those who had the highest ASRS scores, and may warrant additional attention in evaluating and managing their symptoms. Thirdly, mental health assessments and support are important in the care of adults with T1D and ADHD, given the strong association with depression.

Our study did not find strong associations between ADHD symptoms or ADHD medication and cardiometabolic conditions or diabetes-associated liver and kidney damage indicated by any laboratory measures, but these analyses were limited by the small number of individuals with these comorbidities or measures. The link between ADHD symptoms with PVD, although not significant after the corrections, suggests that further study of cardiovascular comorbidities is warranted, since most individuals with PVD have other cardiovascular conditions. It may also reflect a known relationship with smoking reported in both ADHD and PVD [44,45]. Also notable is that most of the previous studies on the associations of ADHD and diabetes-related complications were in youth [12,13,39,41,42]. Given the small sample size in our study and a relatively low percentage of people who were prescribed ADHD medications and had comorbidities, future studies with larger sample sizes are needed to further investigate the impact of ADHD symptoms and the effects of ADHD medications in adults with T1D on the development of both microvascular and macrovascular complications.

In addition to the small sample size, we note that there were several other limitations to our study. First, we had a relatively low survey response rate of 14.1%, which could introduce selection bias if adults with ADHD preferentially responded. Secondly, self-reported data on ADHD symptoms can be further associated with response bias and subjectivity. Thirdly, the cross-sectional design lacked longitudinal insights, so it was not possible to establish causal relationships between ADHD, ADHD medication, and clinical outcomes.

In conclusion, our study highlights the prevalence of ADHD symptoms among adults with T1D and the need for increased recognition and treatment of these symptoms. It underscores the importance of tailored approaches for individuals with T1D, and the integration of mental health support into diabetes care. Further research with larger sample sizes and longitudinal designs is warranted to explore the complex relationships between ADHD, type 1 diabetes, and cardiometabolic outcomes.

## Figures and Tables

**Table 1:** Participant characteristics and ASRA symptoms in ASRS positive and ASRS negative adults with type 1 diabetes.

Total N = 273	ASRS Positive (31.9%; N = 87)	ASRS Negative (68.1%; N = 186)	Statistics
**Age (mean ± SD)**	36.8 ± 14.3	52.5 ± 18.3	*t* = 7.0, *p* < 0.001 *
**Female (N, %)**	63 (72.4%)	114 (61.3%)	χ^2^_(1)_ = 3.2, *p* = 0.07
**White (N = 263)**	81 (93.1%)	182 (97.9%)	χ^2^_(1)_ = 3.8, *p* = 0.052
**Other Race (N = 10)**	6 (6.9%)	4 (2.2%)	
**Non-Hispanic (N = 267)**	83 (95.4%)	184 (98.9%)	χ^2^_(1)_ = 3.4, *p* = 0.06
**Hispanic or Other Ethnicity (N = 6)**	4 (4.6%)	2 (1.1%)	
**BMI (mean ± SD (N))**	30.4 ± 7.1 (61)	28.9 ± 7.2 (161)	*t* = −1.4, *p* = 0.2
**SES (N, %)**	16 (18.4%)	14 (7.5%)	χ^2^_(1)_ = 7.2, *p* = 0.007
**Smoking (N, %)**	9 (10.3%)	11 (5.9%)	χ^2^_(1)_ = 2.1, *p* = 0.3
**Health Insurance Type (N, %)**			
**Commercial/Private**	34 (39.1%)	58 (31.2%)	χ^2^_(1)_ = 6.4, *p* = 0.09
**Government**	23 (26.4%)	65 (34.9%)	
**Managed Care**	24 (27.6%)	59 (31.7%)	
**Others**	6 (6.9%)	4 (2.2%)	
**ASRS Symptom Counts (Mean ± SD)**			
**Total ADHD Symptoms**	10.6 ± 3.0	1.65 ± 2.1	
**Inattention**	6.2 ± 1.9	0.8 ± 1.2	
**Hyperactivity/Impulsivity**	4.3 ± 2.3	0.8 ± 1.2	
**Emotional Dyscontrol**	2.3 ± 1.2	0.6 ± 1.1	χ^2^_(1)_ = 220.7, *p* < 0.0001
**Executive Dysfunction**	5.0 ± 2.4	0.9 ± 1.4	χ^2^ _(1)_ = 119.8, *p* < 0.0001
**ADHD diagnosis and medications (N, % in either ASRS positive or negative groups**			
**Having ADHD Dx (N, %)**	10 (11.5%)	4 (2.2%)	χ^2^_(1)_ = 10.6, *p* = 0.001
**Having ADHD Med (N, %)**	23 (26.4%)	18 (9.7%)	χ^2^_(1)_ = 13.0, *p* < 0.0001
**Having either ADHD Dx or Med (N, %)**	24 (27.6%)	18 (9.7%)	χ^2^_(1)_ = 14.6, *p* < 0.0001

**Note: SD—standard deviation; Dx—diagnosis; Med—medication. SES**: Had any of these recorded socioeconomic stress in the past 12 months: financial strain, housing and transportation problems, and food insecurity. * significant after Bonferroni correction.

**Table 2:** Cardiometabolic comorbidities in ASRS positive and ASRS negative groups.

	ASRS Positive	ASRS Negative	OR	Difference Between the Two Groups *
**Total N = 273**	**(n, %)**	**(n, %)**	**(95%CI)**	
**Obesity ^#^**	31 (35.6%)	64 (34.4%)	1.1 (0.6, 2.1)	χ^2^_(1)_ = 0.1, *p* = 0.7
**Hypertension**	21 (24.1%)	96 (51.6%)	0.8 (0.4, 1.6)	χ^2^_(1)_ = 1.2, *p* = 0.3
**Cardiovascular Diseases**	18 (20.7%)	60 (32.3%)	1.4 (0.7, 3.1)	χ^2^_(1)_ = 0.3, *p* = 0.6
Acute Coronary Syndrome	1 (1.1%)	8 (4.3%)	1.0 (0.1, 13.7)	χ^2^_(1)_ = 0.4, *p* = 0.5
Acute Myocardial Infarction	0 (0.0%)	8 (4.3%)		N/A
Any Ischemic Heart Disease	6 (6.9%)	23 (12.4%)	2.1 (0.6, 7.0)	χ^2^_(1)_ = 0.5, *p* = 0.5
Arrythmia (Broad)	2 (2.3%)	20 (10.8%)	0.5 (0.1, 2.7)	χ^2^_(1)_ = 1.2, *p* = 0.3
Atrial Fibrillation or Flutter	0 (0.0%)	6 (3.2%)		N/A
Cardiac Arrest	0 (0.0%)	0 (0.0%)		N/A
Deep Vein Thrombosis	2 (2.3%)	3 (1.6%)	1.0 (0.1, 10.8)	χ^2^_(1)_ = 0.1, *p* = 0.8
Heart Disease or Heart Failure	8 (9.2%)	29 (15.6%)	2.3 (0.7, 6.9)	χ^2^_(1)_ = 1.0, *p* = 0.3
Peripheral Artery Disease	4 (4.6%)	9 (4.8%)	3.5 (0.7, 17.9)	χ^2^_(1)_ = 2.6, *p* = 0.1
**Peripheral Vascular Diseases**	**9 (10.3%)**	**14 (7.5%)**	**3.8 (1.2, 12.5)**	**χ^2^_(1)_ = 5.2, *p* = 0.02**
Pulmonary Emboli	0 (0.0%)	3 (1.6%)		N/A
Supraventricular Tachycardia	0 (0.0%)	2 (1.1%)		N/A
Ventricular Tachycardia	0 (0.0%)	1 (0.5%)		N/A
Any Cerebrovascular Diseases	3 (3.4%)	15 (8.1%)	1.4 (0.2, 8.4)	χ^2^_(1)_ = 0.1, *p* = 0.8
Subarachnoidal Bleeding	0 (0.0%)	0 (0.0%)		N/A
Ischemic Stroke	3 (3.4%)	4 (2.2%)	2.8 (0.3, 24.5)	χ^2^_(1)_ = 1.4, *p* = 0.2
Hemorrhagic Stroke	0 (0.0%)	0 (0.0%)		N/A

^#^ Had/have a diagnosis of obesity or last BMI ≥ 30 kg/m^2^; * adjusted by race, gender, ethnicity, age, socioeconomic stress, and smoking status. Note: peripheral vascular diseases were not significant after the Bonferroni correction.

**Table 3:** Laboratory and mental health measures in ASRS positive and ASRS negative groups.

	ASRS Positive		ASRS Negative		
Measurements Abnormal Range	N	Mean ± SD %	N	Mean ±SD %	Group Difference
**HbA1c**	85	8.3 ± 1.7	185	8.2 ± 10	χ^2^_(1)_ = 0.2, *p* = 0.6
**High-A1c (≥8.0%)**	45	52.9%	47	25.4%	χ^2^_(1)_ = 6.2, *p* < 0.0001 *
**ALT**	83	19.4 ± 10.4	180	23.4 ± 15.7	χ^2^_(1)_ = 0.4, *p* = 0.5
**High-ALT (>56 U/L)**	2	2.4%	6	3.3%	χ^2^_(1)_ = 0.2, *p* = 0.7
**Creatinine**	84	0.8 ± 0.2	181	1.0 ± 0.5	χ^2^_(1)_ = 14.9, *p* < 0.0001 *
**High-Creatinine**	19	10.5%	2	2.4%	χ^2^_(1)_ = 5.2, *p* = 0.02
**eGFR (mL/min/1.73 m^2^)**	53	81.2 ± 23.5	150	76.3 ± 18.2	χ^2^_(1)_ = 0.2, *p* = 0.7
**Low-eGFR (<60)**	4	7.5%	24	16%	χ^2^_(1)_ = 2.4, *p* = 0.1
**LDL-Cholesterol (mg/dL)**	82	97.2 ± 33.6	181	87.3 ± 30	χ^2^_(1)_ = 1, *p* = 0.3
**High-LDL (≥130)**	13	15.9%	12	6.63%	χ^2^_(1)_ = 5.6, *p* = 0.02
**Triglycerides (mg/dL)**	82	96.5 ± 60.6	182	94.3 ± 59.9	χ^2^_(1)_ = 0, *p* = 0.8
**High-TG (** **≥** **200)**	4	4.9%	10	5.5%	χ^2^_(1)_ = 0.04, *p* = 0.8
**PHQ-2 score**	86	0.5 ± 1.2	185	0.2 ± 0.6	χ^2^_(1)_ = 2.1, *p* = 0.2
**PHQ-9 score**	46	10 ± 7.3	49	6.1 ± 6	χ^2^_(1)_ = 9.2, *p* = 0.002 *

Note: * Creatinine normal range is 0.7 to 1.3 mg/dL (61.9 to 114.9 µmol/L) for men and 0.6 to 1.1 mg/dL (53 to 97.2 µmol/L) for women. High creatinine was defined as above the normal range corresponding to men and women. TG, triglycerides.

## Data Availability

The patient data used in this study cannot be made publicly available. However, summary results and aggregated findings are presented in the manuscript. Additional details may be available upon request and subject to institutional and ethical review board approval.

## Supplementary Materials

The following supporting information can be downloaded at: https://www.mdpi.com/article/10.3390/jcm14103606/s1, Table S1: Association of ASRS total and sub-scores with cardiometabolic conditions; Table S2: Association of ASRS total and sub-scores with laboratory measures and PHQ scores. Figure S1: ASRS symptom counts and HbA1c levels were positively correlated (Spearman’s r = 0.28, *p* < 0.0001).

## Abbreviations

The following abbreviations are used in this manuscript:

T1D	Type 1 diabetes mellitus
T2D	Type 2 diabetes
ASRS	The Adult Self-Report Scale V1.1
ADHD	Attention-deficit/hyperactivity disorder
DKA	Diabetic ketoacidosis
PHQ	Patient Health Questionnaire
Glm	Generalized linear regression
PVD	Peripheral vascular disease
