# Supplementary material for: Attention-Deficit/Hyperactivity Disorder Symptoms Are Common and Associated with Worse Glycemic Control in Adults with Type 1 Diabetes
Source: J Clin Med. 2025 May 21;14(10):3606. doi: 10.3390/jcm14103606 (PMC12112195; doi:10.3390/jcm14103606)
Supplement: Supplementary file 1 [file jcm-14-03606-s001.zip › jcm-3598887-supplementary Figure.pdf]

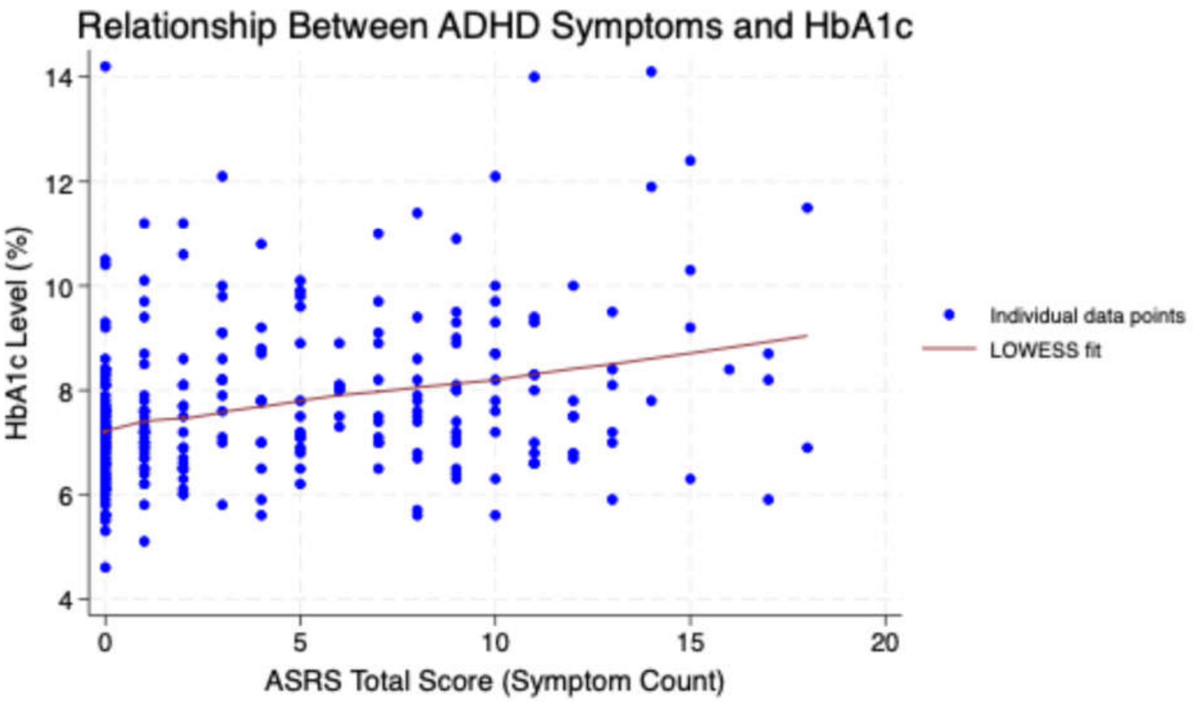

Supplementary Figure S1. ASRS symptom counts and HbA1c levels were positively correlated (Spearman's r = 0.28, p<0.0001)
