# Supplementary material for: Attention-Deficit/Hyperactivity Disorder Symptoms Are Common and Associated with Worse Glycemic Control in Adults with Type 1 Diabetes
Source: J Clin Med. 2025 May 21;14(10):3606. doi: 10.3390/jcm14103606 (PMC12112195; doi:10.3390/jcm14103606)
Supplement: Supplementary file 1 [file jcm-14-03606-s001.zip › Supplementary Tables.pdf]

**Supplementary Table S1. Association of ASRS total and sub-scores with cardiometabolic conditions.**

| Diseases | Total ADHD Scores | | Hyperactivity /Impulsivity | | Inattention | | Emotional Dyscontrol | | Executive Dysfunction | |
|---|---|---|---|---|---|---|---|---|---|---|
| | $\chi^2_{(1)}$ | p values | $\chi^2_{(1)}$ | p values | $\chi^2_{(1)}$ | p values | $\chi^2_{(1)}$ | p values | $\chi^2_{(1)}$ | p values |
| **Obesity** | 0.03 | 0.852 | 1.62 | 0.204 | 0.24 | 0.625 | 2.1 | 0.147 | 0.42 | 0.518 |
| **Hypertension** | 1.12 | 0.291 | 1.35 | 0.245 | 0.7 | 0.403 | 3.73 | 0.054 | 0.01 | 0.938 |
| **Cardiovascular Diseases** | 0.44 | 0.507 | 0.4 | 0.527 | 0.10 | 0.76 | 0.1 | 0.783 | 1.2 | 0.274 |
| Acute Coronary Syndrome | 0.47 | 0.495 | 0.36 | 0.55 | 1.35 | 0.246 | 0.9 | 0.344 | 0.07 | 0.791 |
| Acute Myocardial Infarction | | | | | | | | | | |
| Any Ischemic Heart Disease | 0.04 | 0.849 | 1.33 | 0.248 | 1.49 | 0.222 | 0 | 0.955 | 0.47 | 0.492 |
| Arrythmia (Broad) | 0 | 0.992 | 0.86 | 0.353 | 0.27 | 0.604 | 0.48 | 0.487 | 0.97 | 0.324 |
| Atrial Fibrillation or Fludder | | | | | | | | | | |
| Cardiac Arrest | | | | | | | | | | |
| Deep Vein Thrombosis | 0.09 | 0.765 | 0.8 | 0.372 | 0.16 | 0.69 | 0.87 | 0.35 | 0.01 | 0.912 |
| Heart Disease or Heart Failure | 0.32 | 0.569 | 0.84 | 0.359 | 0.54 | 0.461 | 0.18 | 0.673 | 0.72 | 0.395 |
| Peripheral Artery Disease | 4.07 | 0.044 | 0.59 | 0.442 | 0.79 | 0.374 | 0 | 0.982 | 0.78 | 0.376 |
| Peripheral Vascular Diseases | **6.86** | **0.009** | 0.02 | 0.881 | 0.72 | 0.398 | 0 | 0.969 | 0.75 | 0.385 |
| Pulmonary Emboli | | | | | | | | | | |
| Supraventricular Tachycardia | | | | | | | | | | |
| Ventricular Tachycardia | | | | | | | | | | |
| Any Cerebrovascular Diseases | 0.17 | 0.682 | 0 | 0.953 | 0.02 | 0.893 | 0.33 | 0.568 | 0.02 | 0.88 |
| Subarachnoidal Bleeding | | | | | | | | | | |
| Ischemic Stroke | 1.52 | 0.218 | 0.37 | 0.543 | 2 | 0.158 | 1.86 | 0.173 | 0.21 | 0.646 |
| Hemorrhagic Stroke | | | | | | | | | | |

**Supplementary Table S2. Association of ASRS total and sub-scores with laboratory measures and PHQ scores.**

| | Total Scores | | Hyperactivity /Impulsivity | | Inattention | | Emotional Dyscontrol | | Executive Dysfunction | |
|---|---|---|---|---|---|---|---|---|---|---|
| **Measurements** | $\chi^2_{(1)}$ | **p values** | $\chi^2_{(1)}$ | **p values** | $\chi^2_{(1)}$ | **p values** | $\chi^2_{(1)}$ | **p values** | $\chi^2_{(1)}$ | **p values** |
| HbA1c | 0.4 | 0.527 | 0.8 | 0.384 | 0 | 0.96 | 2.5 | 0.116 | 0.4 | 0.519 |
| ALT | 0.3 | 0.604 | 0 | 0.995 | 0.7 | 0.412 | 2.6 | 0.11 | 0.2 | 0.659 |
| Creatinine | 3.5 | 0.063 | 1.2 | 0.276 | 0.1 | 0.716 | 0 | 0.838 | 0.6 | 0.429 |
| eGFR | 0.8 | 0.386 | 0.4 | 0.516 | 0.1 | 0.701 | 1.4 | 0.245 | 0 | 0.948 |
| LDL-Cholesterol | 0 | 0.939 | 1.6 | 0.201 | 1 | 0.325 | 0.2 | 0.693 | 6.2 | **0.013** |
| Triglycerides | 0 | 0.875 | 0 | 0.964 | 0.1 | 0.787 | 7.4 | 0.006 | 2 | 0.16 |
| PHQ-2 | 2.6 | 0.11 | 0.4 | 0.542 | 0.1 | 0.793 | 2.5 | 0.113 | 2.5 | 0.115 |
| PHQ-9 | 11.7 | **0.0006*** | 3.2 | 0.073 | 0.1 | 0.814 | 5.1 | **0.023** | 0.2 | 0.625 |

Note: * Significant after multiple corrections
